# Atomic Force Microscopy Analysis of Velocity Dependent Adhesive Viscoelastic Contact

**DOI:** 10.1021/acs.langmuir.4c03370

**Published:** 2024-11-08

**Authors:** Nobuhito Onozuka, Ken Nakajima

**Affiliations:** School of Materials and Chemical Technology, Tokyo Institute of Technology, 2-12-1, O-Okayama, Meguro-ku, Tokyo 152-8552, Japan

## Abstract

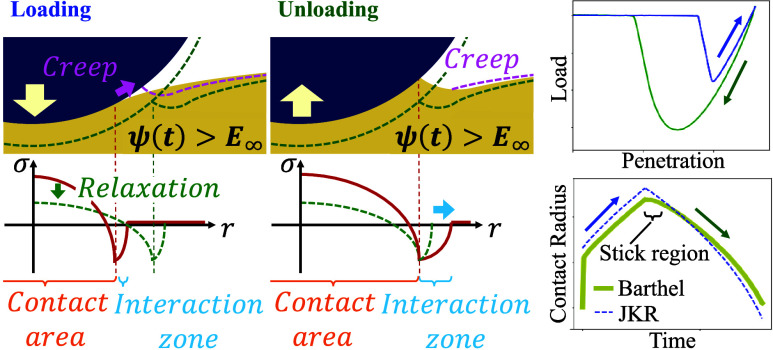

Adhesive contact phenomena play a crucial role in various scientific and engineering fields. However, considering viscoelasticity, which is essential for understanding practical applications involving soft materials like polymers, makes analysis challenging. Traditional elastic contact models such as the Johnson–Kendall–Roberts and Maugis–Dugdale models often fail to account for viscoelastic behavior. In this study, rate-dependent viscoelastic adhesive contacts were analyzed using atomic force microscopy force–distance curve measurements, comparing the elastic models with the viscoelastic model proposed by Barthel. The force curve analysis, conducted with the Barthel model for the first time, reveals that viscoelastic behaviors inside the contact area and the interaction zone both affect the contact state. These viscoelastic behaviors result in phenomena specific to viscoelastic contact, such as the “stick region” and the apparent work of adhesion. The Barthel model successfully captures the rate dependence of the contact situation, promoting a comprehensive understanding of viscoelastic adhesive contact phenomena.

## Introduction

The contact phenomena are involved in many areas of science and engineering, but of particular interest are those accompanying adhesion.^1−5^ Adhesion is especially noticeable at microscopic scales where the surface-to-bulk ratio is large and attractive surface forces become non-negligible. Therefore, further understanding of adhesive contact requires both theoretical and experimental investigation of microscopic contact, given that macroscopic contact can be considered to consist of numerous microscopic asperities.

Such microscale contact phenomena can be experimentally studied using instruments like the surface force apparatus^6,7^ or the atomic force microscope (AFM).^8,9^ AFM is often employed to gain a detailed understanding of nanoscale contact.^9−16^ Force–distance curve measurements, in particular, are commonly used to easily obtain the load and penetration within microscopic, possibly single asperity, contacts.^11−13^ Since the contact radius in AFM is too small to be directly measured, it must be estimated by analyzing the measured data with some contact mechanics models for the single asperity contact problem.

The effect of adhesion on the contact model depends on the degree of additional deformation caused by the adhesion force, which is influenced not only by the strength of the adhesion force but also by the size and softness of the bodies involved in the contact. If the deformation caused by the adhesion force is small—i.e., the adhesion force is weak, the contact radius is large, and the body is stiff—such contact is usually explained by the Derjaguin–Muller–Toporov (DMT) model.^17^ Conversely, if the deformation caused by the adhesion force is large—i.e., the adhesion force is strong, the contact radius is small, and the body is compliant—such contact is often represented by the Johnson–Kendall–Roberts (JKR) model.^18^ These are both extreme models of adhesive contact, and actual contact is positioned in an intermediate state between these models. This intermediate situation (the JKR–DMT transition) was first described in a unified manner by Tabor,^19^ after which Maugis proposed an analytical model [the Maugis–Dugdale (MD) model].^20^ The double-Hertz model has also been proposed by Greenwood and Johnson as a model that can represent the JKR–DMT transition.^21^ The difference between these models lies in how they consider the region where the adhesion force acts, often called the interaction zone.

Unfortunately, since these contact models have all started with the Hertz model,^22^ which deals with elastic materials, they cannot explain cases involving viscoelastic soft materials such as polymers. In the case of viscoelastic contact, the softness that determines the contact changes with the time scale over which the contact occurs, making the contact phenomenon rate-dependent and difficult to handle.

Some attempts have been made to explain this rate dependency by incorporating the idea of fracture mechanics.^23−27^ Maugis and Barquins, for example, have shown that the apparent work of adhesion, i.e., the energy required to move the contact line, exhibits rate dependence in the case of viscoelastic materials.^23^ Greenwood theoretically explained this behavior by considering rate-dependent contact edge shapes,^24−26^ based on earlier results provided by Schapery.^27^ These attempts suggest that the key to explaining such rate-dependent contacts is to take into account the viscoelastic behavior near the contact line, i.e., in the interaction zone, within the contact model.

In this regard, several contact models have attempted to represent viscoelastic contact by separating the interaction zone and the rest of the region (i.e., bulk scale).^24,28−35^ In those models, the contact process at the bulk scale is considered slow enough compared to the relaxation of the viscoelastic materials and is therefore represented with elastic models under a fully relaxed state, while that in the interaction zone is considered to occur more instantaneously and is expressed with a rate-dependent under-relaxed state. In the sense that the viscoelastic rate dependence is attributed only to the interaction zone, such models can be described as “quasi-viscoelastic” contact models. A more direct model for viscoelastic contact has been proposed by Barthel et al.^36−38^ They expressed the rate dependence of the adhesive viscoelastic contact by extending the definitive solution of the viscoelastic contact without adhesion proposed by Ting,^39^ using the interaction zone based on the double-Hertz model. There are also attempts to represent viscoelastic contact using the finite element method, though it is more complex and difficult to handle.^40,41^

Studies on the theoretical side have been conducted in this way; however, attempts to link these theories to actual experimental data are still lacking, especially for the viscoelastic adhesive contact. There have been several studies of “quasi-viscoelastic” contact models that have been compared to experiments,^32−35^ but there are no such examples of more direct viscoelastic contact models like the Barthel model, perhaps due to its complex representation.

In this article, viscoelastic adhesive contacts were investigated using AFM force–distance curve measurements. These were analyzed with the viscoelastic Barthel model and then compared with results based on the elastic contact models, specifically, the JKR and MD models. To the best of our knowledge, force curve analysis using the Barthel model has been performed for the first time, enabling a unified discussion of the rate dependence of contact behavior in both loading and unloading processes from experimental and theoretical perspectives. For measurements at sufficiently slow ramp rates, an elastic force curve where the loading and unloading curves overlapped was obtained, and the contact radii estimated by the elastic models and the Barthel model agreed well. This indicates that such contact can be explained in a fully relaxed state, and at the same time suggests that the analysis using the Barthel model can also handle the elastic contact. As the ramp rate increased, hysteresis of the force curve due to viscoelastic losses was observed, and the contact radius estimated from the Barthel model differed from that of the elastic models, implying that the contact can no longer be considered to be occurring in the fully relaxed state. The Barthel model allows for the representation of the peculiar behavior of viscoelastic contact, for example, the stick region. Further analysis suggested that deviations from the elastic model at high rates can be explained by viscoelastic behavior near and inside the contact line, and the subsequent apparent work of adhesion.

## Theory

### Adhesionless Elastic Contact

The concept of elastic contact without adhesion was first formulated by Hertz,^22^ and later generalized for arbitrary bodies by Sneddon.^42^ Sneddon’s analytical equations for the applied load *P* and the penetration δ can be expressed as follows
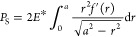
1
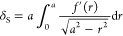
2

The equations include *a*, the contact radius; *f*(*x*), the expression of the indenter shape; and E*, the reduced modulus. The reduced modulus is defined as E* ≡ *E*/(1 – ν^2^), where *E* and ν are the Young’s modulus and the Poisson’s ratio, respectively. Equation 2 indicates that the penetration is geometrically determined from the indenter shape and the contact radius. The integrand in eq 1 can be related to the penetration by the following relation
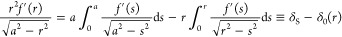
3where δ_0_(*r*) can be interpreted as the Hertz-like penetration when the contact radius is *r*. Therefore, eq 1 can be transformed using eq 3 as follows
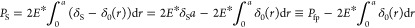
1′

The first term represents the load required to push in δ_S_ under a constant contact radius *a* (i.e., the applied force for flat punch contact), and the second term accounts for the excess force considered due to the difference between the flat punch shape and the actual indenter shape.

The well-known Hertz’s solution can be derived by considering a parabolic indenter whose apex curvature radius is *R* in Sneddon’s expression. In this case, δ_0_(*r*) = *r*^2^/*R*, then
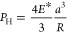
4
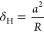
5

### Adhesive Elastic Contact

As already mentioned, there are two fundamental theories for adhesive elastic contact: the DMT model^17^ and the JKR model.^18^

The DMT model assumes that adhesion does not affect the deformation of the sample, i.e., the Hertzian deformation is maintained, though the load due to the adhesion force is added to the applied force. Therefore, eq 5 is left as it is, and eq 4 is modified as follows, using the work of adhesion *w*
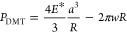
6

The 2π*wR* term that is added to eq 4 represents the effect of the adhesion force.

In contrast, the JKR model accounts for additional sample deformation caused by adhesion by considering the energy balance between the change in mechanical energy associated with the deformation exerted by the adhesion and the surface energy derived from that adhesion. The resulting applied load and penetration are then specified as follows
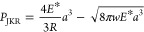
7
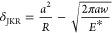
8

There was a controversy since these models yielded different results for the same system, until Tabor explained that these two models represented both extremes of the contact condition, varying in scale and softness.^19^ Tabor introduced a parameter known as Tabor’s parameter
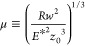
9where *z*_0_ is the equilibrium separation of the surface. The DMT model is effective in the limit of μ ≪ 1 (i.e., for small *R* and large E*) while the JKR model is effective in the limit of μ ≫ 1 (i.e., for large *R* and small E*). The actual adhesive elastic contact occurs at an intermediate state between these models.

In this context, Maugis proposed the MD model,^20^ which described the JKR–DMT transition by introducing the step-shaped Dugdale potential^43^ to represent the adhesion force distribution near the contact line. The MD model consists of following four equations. Using normalized values, , , and , the equations are
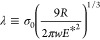
10
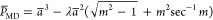
11
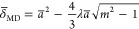
12
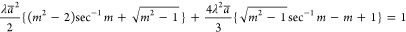
13

The introduced parameter λ is equivalent to Tabor’s parameter (λ ≈ 1.16 μ)^44^ and determines the JKR–DMT transition state. Since σ_0_ is a positive value representing the maximum adhesive stress predicted by the Lennard-Jones potential (σ_0_ ≈ 1.03*w*/*z*_0_), eqs 9 and 10 have almost the same physical meaning. Under the Dugdale potential, the adhesive stress acts only within a region in the vicinity of the contact line, *a* ≤ *r* ≤ *c*, and is considered to take a constant value σ_0_. The region where the adhesion force acts is often referred to as the interaction zone. Maugis incorporates this interaction zone into the contact model via *m* defined as *m* ≡ *c*/*a*. This simple treatment of the interaction zone enables the JKR–DMT transition to be expressed analytically.

Greenwood et al. also proposed the double-Hertz model^21^ which serves as an alternative to the MD model. Unlike the MD model, which utilizes the Dugdale potential with a constant adhesive stress region, the double-Hertz model considers its interaction zone by assuming the adhesive stress distribution, σ(*r*), within the interaction zone to be ellipsoidal
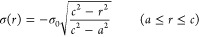
14

If *c* ≤ *r*, then σ(*r*) = 0. Consequently, the penetration and the applied load can be expressed as follows
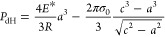
15
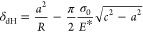
16

The value of *c* can be determined using the following equation, which incorporates Tabor’s parameter
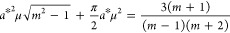
17here,  is represented in a normalized form. It should be noted that eq 17 is similar to eq 13. The results obtained by the double-Hertz model can handle the JKR–DMT transition as effectively as the MD model. However, it is important to point out that the results of the double-Hertz model are expressed using only elementary functions as in eqs 15–17, making them easier to handle compared to the MD model, which utilizes elliptic functions as in eqs 11–13.

From both the MD model and the double-Hertz model, one can derive the DMT and the JKR models as the respective limits of the interaction zone size. The DMT model results when the interaction zone is sufficiently wide relative to the contact area, while the JKR model emerges when the zone is localized at the contact line. In the MD model, eqs 11 and 12 simplify to the DMT model (eqs 5 and 6) as λ → 0, and to the JKR model (eqs 7 and 8) as λ → *∞*. In the double-Hertz model, the JKR–DMT transition is directly controlled by *c*, which in turn is determined by μ.

The representation of the interaction zone is crucial when addressing viscoelastic contact. For instance, in the JKR model, the localization of the interaction zone at the contact line implies that the adhesion force diverges at that point, a scenario that is unrealistic in practical situations. Furthermore, regardless of how slowly the contact condition progresses, only instantaneous deformation is considered at the contact line, precluding any discussion of rate-dependence. Both the MD model and the double-Hertz model address this by considering a finite interaction zone, thereby preventing the divergence of the adhesion force. However, since these models do not account for rate dependency, they still struggle with handling viscoelastic contact effectively. Consequently, to accurately represent viscoelastic contact, contact models that consider both the interaction zone and its rate dependence are essential.

Many force curve analyses still use simple elastic contact models, even when viscoelasticity cannot be ignored during AFM measurements. This paper focuses on this issue and aims to clarify the difference between cases where viscoelastic contact is deliberately analyzed using the elastic contact models and cases where the viscoelastic contact model is used correctly. The JKR and the MD model were used as the elastic contact models, and the details of the analytical approaches are described in Supporting Information.

### Adhesionless Viscoelastic Contact

For the adhesionless case, a definitive solution for viscoelastic contact has been presented by Ting,^39^ based on an extension of Sneddon’s elastic solutions. Considering a single cycle of loading–unloading process, the applied load during both loading and unloading phases can be expressed as follows
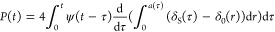
18here, ψ(*t*) represents the relaxation function of the viscoelastic material. This expression models the viscoelastic contact as a convolution of Sneddon’s solution (referenced in eq 1′) under an elastic modulus that relaxes over time since the force was first applied at each position.

Ting’s approach can indeed be extended to more complex loading–unloading histories. However, no examples could be found where this approach is applied directly to force curve analysis. Regarding the loading phase, Johnson proposed a simpler model,^45^ which has been employed in AFM force curve analyses.^46,47^

### Adhesive Viscoelastic Contact

As mentioned earlier, for the adhesive case, the key is the treatment of the interaction zone and its rate dependence. A simple approach is to divide the contact region into the interaction zone and the remaining bulk region. Given that the contact process is sufficiently slower than the viscoelastic relaxation process, the bulk contact can be expressed using elastic contact models under fully relaxed properties. This is not the case for the interaction zone. Drawing an analogy with fracture mechanics, it is expected that the stress and deformation in the vicinity of the contact line exhibit steep changes, even if the contact process is sufficiently slow. Therefore, the interaction zone is considered to be in a rate-dependent, under-relaxed state rather than a fully relaxed state. Based on this idea, some models attempt to describe adhesive viscoelastic contact by attributing the rate dependence solely to the interaction zone.^24,28−35^ In a sense, such models could be called “quasi-viscoelastic” models. Some studies have described the rate dependence of the contact using “quasi-viscoelastic” models and compared these with experimental data.^32−35^ However, those studies focused only on the rate dependence of the unloading process, and none have been analyzed loading and unloading together in a unified manner. Furthermore, it should be noted that these studies assumed the unloading process started from a fully relaxed state. This assumption does not always hold, depending on the loading–unloading history, which limits the application of the “quasi-viscoelastic” models in real cases. More importantly, the assumption that the bulk contact can be represented in a fully relaxed state also needs careful consideration to determine its validity.

For the concerns mentioned above, models that consider the relaxation state at all points involved in the contact is desirable, rather than considering the relaxation state in the interaction zone and the bulk region separately. One such model is the one proposed by Barthel et al.^36−38^

The Barthel model extends Ting’s solution to the adhesive case by combining it with the double-Hertz model to represent the interaction zone. Here, an approximate model for the situation where the interaction zone is sufficiently small compared to the contact radius is described.^37^ In this model, the mechanical equilibrium equation relating stress and deformation reduces to an equation linking the applied load, the penetration, and the contact radius as follows
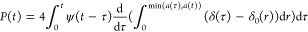
19

Note that this equation has almost the same form as eq 18, but δ(τ) in eq 19 is differs from δ_S_ in eq 18 due to the effects of the adhesion force, and the integral range for *r* is different.

Additionally, to avoid discrepancies between the adhesive stress distribution given by the double-Hertz model (eq 14) and the contact edge shape inside the interaction zone, the following self-consistency equations are introduced
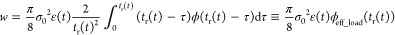
20
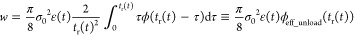
21where eqs 20 is for loading, while eq 21 is for unloading. ε(*t*) ≡ *c*(*t*) – *a*(*t*) is the interaction zone width based on the double-Hertz model, and *t*_r_(*t*) ≡ ε(*t*)/|d*a*(*t*)/d*t*| is the dwell time representing the time required for the contact line to pass through the interaction zone. Since  and  are moments of the creep compliance governed by *t*_r_, they represent the effective creep compliance dominating the behavior inside the interaction zone. Note that eq 19 relates to the interior of the contact area, whereas the self-consistency (eqs 20 and 21) relate to the interaction zone outside the contact area.

To avoid discontinuities or conflicts in the contact state inside and outside the contact area, these equations must be connected by the coupling equations as follows, using the auxiliary function *g*(*a*(*t*),*t*) determined by a given time *t* and the contact line position *a*(*t*) at that time
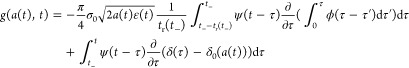
22
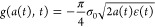
23*t*_–_ denotes the time when the position *a*(*t*) first entered inside the contact area. *g*(*a*(*t*),*t*) is defined as a suitable transform of the normal surface stress, as follows
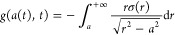
24

Equation 22 is derived from the equilibrium equation and the self-consistency equation. Qualitatively, both the adhesive stress inside the interaction zone (the first term on the right-hand side) and the stress inside the contact area (the second term on the right-hand side) are superimposed based on the history up to the time of interest. Equation 23 comes from the double-Hertz model, which can be easily obtained by substituting the adhesive stress distribution of the double-Hertz model (eq 14) into eq 24. For eq 19 and the self-consistency equations to be coupled, eqs 22 and 23 must match via *g*(*a*(*t*),*t*).

The Barthel model can express the viscoelastic adhesive contact more directly because it does not treat the bulk and the interaction zone independently as in the “quasi-viscoelastic” models. However, perhaps due to the complexity of the model, it has not been used for real data analysis. In this article, the Barthel model, which is difficult to handle, was applied to force curve analysis for the first time by combining inductive computation and optimization methods. Detailed procedures are described in the Supporting Information.

## Experimental Section

### Materials

Polydimethylsiloxane (PDMS) was mainly used to evaluate the effect of viscoelasticity on the contact in various contact models. The PDMS sample was prepared by mixing primer (KE-106, Shin-Etsu Chemical, Tokyo) and cross-linker (CAT-RG, Shin-Etsu Chemical, Tokyo) at a ratio of 10:1, defoaming, pouring into a mold, and heating at 75 °C for 1 h and 150 °C for 30 min. Its *T*_g_ and tan δ, measured with an AR2000ex rotational rheometer (TA Instruments, New Castle DE), were −125 °C and 0.02 at 20 °C at 1 Hz, respectively, suggesting that the viscoelasticity of the PDMS was insignificant.

Styrene–butadiene rubber (SBR) provided by Yokohama Rubber (Kanagawa) was also used as a sample with higher viscoelasticity. The *T*_g_ and tan δ of the SBR were −35 °C and 0.36, respectively, indicating that it exhibits greater viscoelasticity than the PDMS.

For the AFM measurements, these samples were cut at −120 °C with an ultramicrotome (EM UC6, Leica Microsystems, Wetzlar).

### Force–Distance Curve Measurements

**Figure 1:**
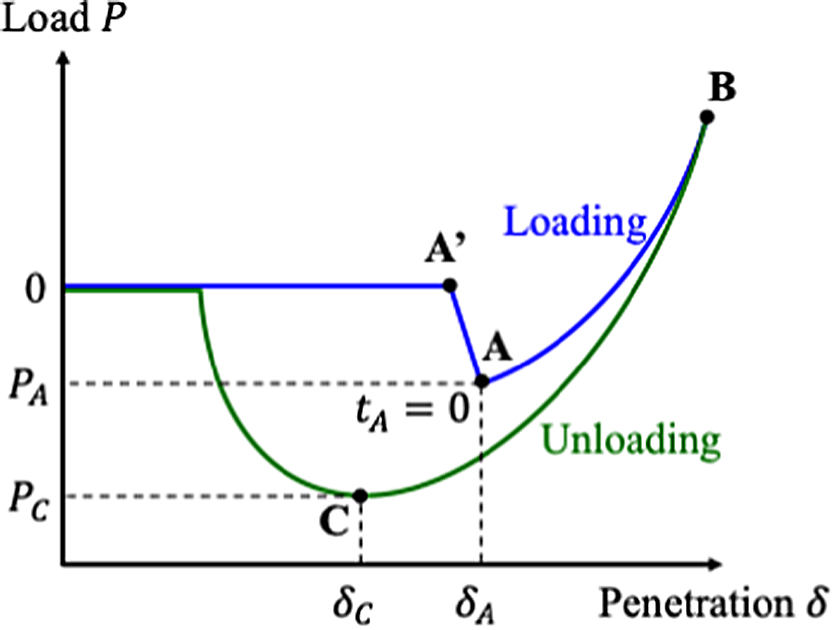
A schematic diagram of the force curve.

The force–distance curve measurement of AFM can easily obtain the relationship between the applied load *P* and the penetration δ (i.e., the force curve) for the microscopic contact. What is obtained during the actual measurement is the deflection of the cantilever *d* and the z-position of the scanner *z*. The applied load is calculated as *P* = *kd* using the spring constant *k* calibrated in advance, and the penetration is calculated as δ = *z* – *d*. Figure 1 shows a typical force curve for a sample with adhesion. As the cantilever tip approaches the sample surface, a jump-in behavior occurs at a certain area (point A′ ∼ A), followed by the tip being pushed into the sample to reach the maximum load point (point B). The cantilever is then retracted from this point until a jump-out behavior occurs after some negative deformation δ_C_ due to the effect of adhesion force (point C). Since the contact radius and the mechanical properties cannot be measured directly, it is necessary to analyze the force curve with contact mechanics to estimate these values.

It is important to note that in the case of a force curve like in Figure 1, its viscoelasticity is already non-negligible, because if the contact were perfectly elastic, the loading and unloading curves would overlap completely. The difference between the two curves when they do not overlap corresponds to the viscoelastic loss.^33^ Although such viscoelastic loss behavior is frequently observed, it is common practice to use elastic contact models even for such curves. Therefore, it is necessary to deepen our understanding in this aspect by comparing the analysis based on elastic contact models with those based on viscoelastic contact models.

The difficulty here is how to handle the zero-point of the penetration, i.e., when the tip makes contact with the surface. The jump-in behavior starts where the gradient of the attractive force acting between the surface and the tip exceeds the spring constant of the cantilever.^48,49^ If the long-range attractive force is negligible, the tip makes contact at point A′, thus this point is regarded as the zero-point. The tip is then pulled in by adhesive interaction to reach point A. However, since the attractive force is not always negligible, the cantilever starts bending before making the contact, thus the tip actually makes contact somewhere between point A′ and A. Furthermore, it is also a question of whether the moment of contact can be regarded as δ = 0, as the sample surface can be negatively deformed by the attractive force. The validity of the zero-point cannot be discussed in usual elastic contact models, so in many cases, it is common to consider point A′ or A as δ = 0, or to take an approach that does not depend on the δ = 0 point.^48,50^ Point A will be referred to as the “jump-in point” throughout this paper; however, note that this point is not necessarily the zero-point of penetration. The introduction of the Barthel model may provide a different approach to this issue, which will be discussed later.

### AFM Measurements

All AFM data were obtained using a Dimension ICON with a NanoScope VI controller (Bruker Nano Surface, Santa Barbara, CA) at room temperature and humidity (about 20 °C, 25%). Note that it has been pointed out that the effect of humidity on the force curve (i.e., capillary force) can be neglected in the case of hydrophobic polymers.^51^ The Dimension ICON has a closed-loop scanner that allows precise determination of the scanner’s *z*-position during force curve measurements, making it suitable for accurate discussion of contact conditions. An LRCH 500 silicon cantilever (Team Nanotec, Villingen-Schwenningen) with a spring constant of 5.01 N/m (calibrated with the thermal fluctuation method^52^) and a tip radius of 625 nm (obtained from the SEM images provided by the manufacturer) was used.

To investigate the viscoelastic rate dependence of each sample, force curves were measured at various ramp rates (10, 30, 301, 3050, and 9770 nm/s). Each force curve was obtained at a random point on the sample surface. Since there was almost no dependence on the measurement point for the force curves, it can be said that the measurements were performed stably and that the sample surfaces were sufficiently uniform.

## Results

### Force Curve Measurements

**Figure 2:**
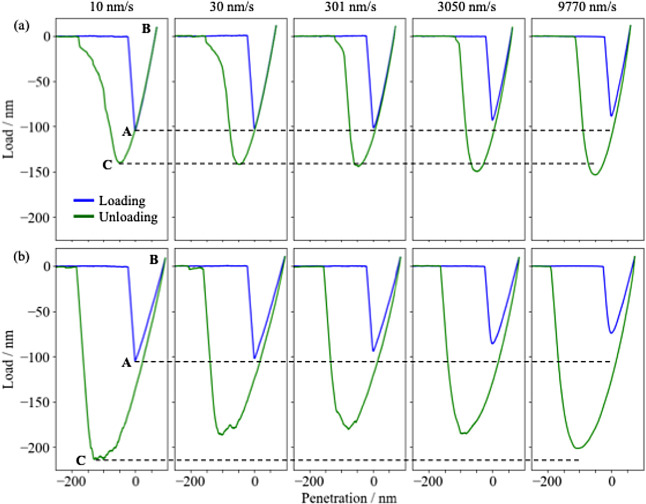
Rate-dependent force curves of (a) the PDMS and (b) the SBR. Blue and green lines correspond to the loading and unloading curves, respectively. Dashed lines indicate the load at points A and C for the smallest ramp rate.

The force curves of the PDMS and the SBR measured by varying ramp rates are shown in Figure 2a,b, respectively. The zero-point of the penetration is taken here at point A for now.

For the PDMS, its loading and unloading curves overlapped at lower ramp rates, indicating that the force curves obtained can be regarded as fully relaxed elastic curves. At higher ramp rates, on the other hand, those curves did not overlap, suggesting that the viscoelasticity is not negligible, even for the PDMS with a fairly small tan δ of 0.02. The pull-off force *P*_C_ at point C was negatively large at higher rates and gradually converged to a constant value at lower rates. This tendency is consistent with the rate dependence of the apparent work of adhesion discussed by Greenwood based on the standard linear solid (SLS) model.^24−26^ A rate dependence was also observed in the load *P*_A_ at point A. This indicates that the degree to which the tip is pulled into the sample at jump-in varies with the time scale of the measurement; i.e., the higher the rate, the more imperfect the tip is pulled in due to the viscoelastic effect. It has been pointed out that in the loading phase of the adhesive contact, it takes a considerably long time to reach equilibrium, even for a sample that equilibrates instantaneously in a bulk mechanical measurement.^53,54^ This is assumed to be because sufficient time is needed for the cantilever tip and elastomer surface to achieve a complete equilibrium adhesion interface when even microscopic roughness is taken into account. The rate dependence of the jump-in observed here may reflect this point of view.

For the SBR, its behavior was more complex than the PDMS. First, its loading and unloading curves did not overlap even at 10 nm/s, indicating that even in this slow rate range, a completely relaxed elastic situation could not be obtained and viscoelastic effects remained. Second, though its jump-in behavior was similar to that of the PDMS, its jump-off behavior could not be interpreted in the same way as the PDMS. There was no convergence of *P*_C_ toward lower rates as seen in the PDMS; rather, it tended to increase negatively in the lower rate range, i.e., it did not behave like the Greenwood case. As mentioned above, Greenwood treated the rate dependence of the apparent work of adhesion based on the SLS model, whose relaxation behavior is expressed in terms of a single relaxation time. However, since the relaxation behavior of actual elastomers consists of multiple relaxation phenomena, there are limitations in expressing their true relaxation with a single relaxation time. The viscoelasticity of the PDMS and the SBR in this experiment is assumed to be mainly due to the glass transition. However, since the glass transition itself cannot be explained by a single relaxation process, it is not strictly appropriate to represent it using the SLS model. The fact that Greenwood-like rate dependency was observed for the PDMS but not for the SBR may suggest that the PDMS, with its sufficiently low *T*_g_ and small viscoelasticity, approximately follows the SLS model under the measurement conditions, whereas the SBR, with its relatively high *T*_g_ and large viscoelasticity, cannot be approximated by the SLS model. Another possibility is that, as viscoelasticity increases, the effect of microscopic roughness on the contact conditions during loading may no longer be negligible, thereby affecting the unloading behavior. Since it has been observed that the adhesion of elastomers is influenced by contact time under microscopic roughness,^54^ it is possible that the adhesion energy during the unloading phase becomes larger due to a longer contact time at a smaller ramp rate.

Such contributions of multiple relaxation phenomena or roughness to the viscoelastic contact have not been adequately studied. Even the viscoelastic Barthel model considered in this paper uses the SLS model with a smooth surface, making it inapplicable to samples showing complex viscoelastic behavior such as the SBR. Therefore, in the following sections, the PDMS, which exhibits simple and tractable viscoelastic behavior, was analyzed with various contact mechanics. Of course, the treatment of complex contact behavior involving multiple relaxation phenomena or roughness needs further research, which is a subject for future research.

### Force Curve Analyses

In the following sections, the contact radius, the interaction zone, and the apparent work of adhesion during the force curve measurements of the PDMS were analyzed from the elastic models (the JKR and the MD) and the viscoelastic Barthel model. Details of the fitting process are provided in Supporting Information, and this section focuses on the differences between models.

### The Contact Radius

**Figure 3:**
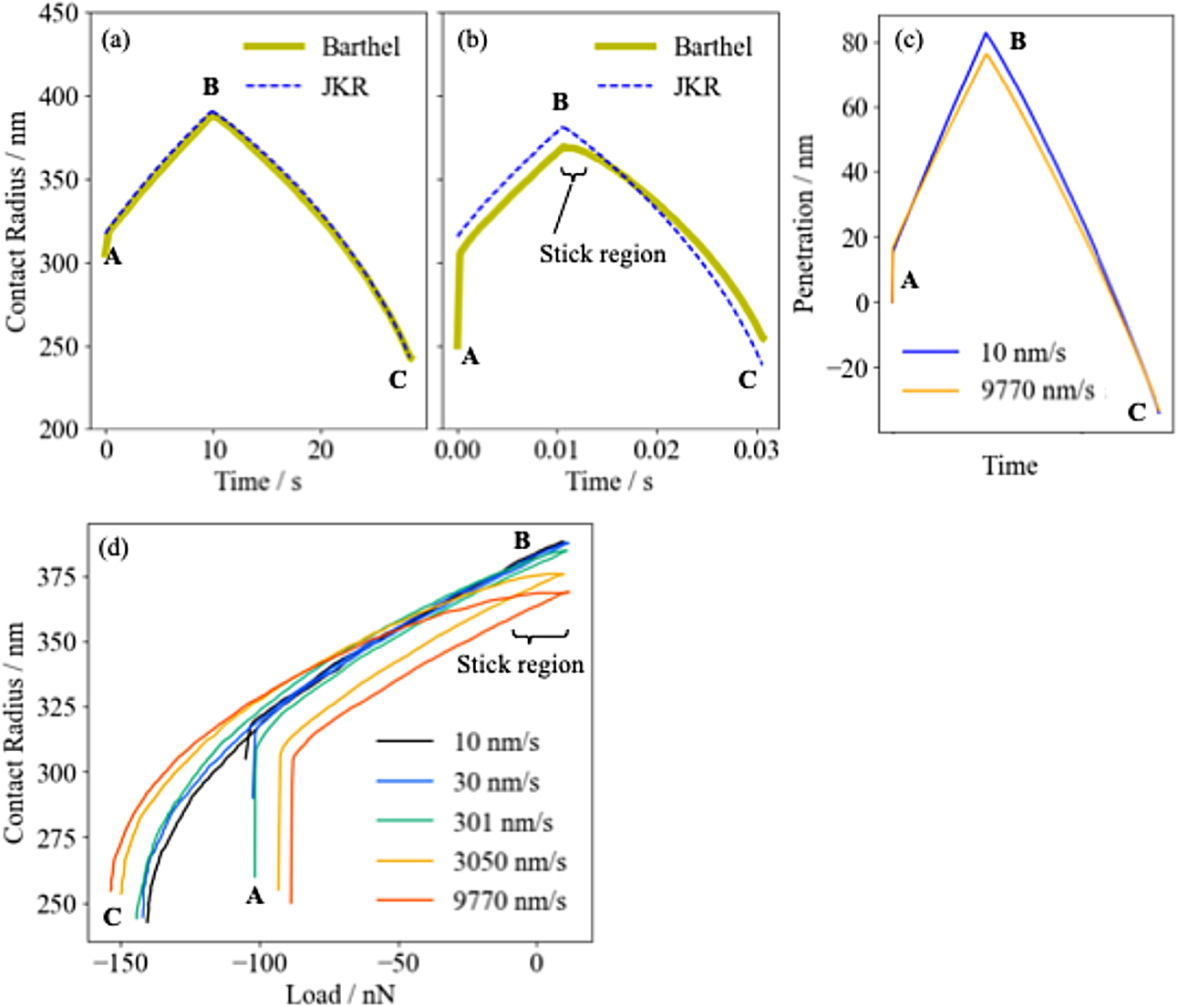
Time variation of the contact radius for the ramp rate of (a) 10 and (b) 9770 nm/s. Thick solid yellow lines and thin dashed blue lines represent the contact radius from the Barthel and JKR models, respectively. (c) shows the time variation of the penetration corresponding to (a,b). (d) shows the relationship between load and contact radius under various ramp rates.

Figure 3a,b show the time variation of the contact radius obtained by the Barthel model analysis of the force curve of the PDMS at a ramp rate of 10 and 9770 nm/s, respectively. The penetration at each ramp rate is shown in Figure 3c. The relationship between load and contact radius for all force curve measurements (10, 30, 301, 3050, and 9770 nm/s) is summarized in Figure 3d.

**Figure 4:**
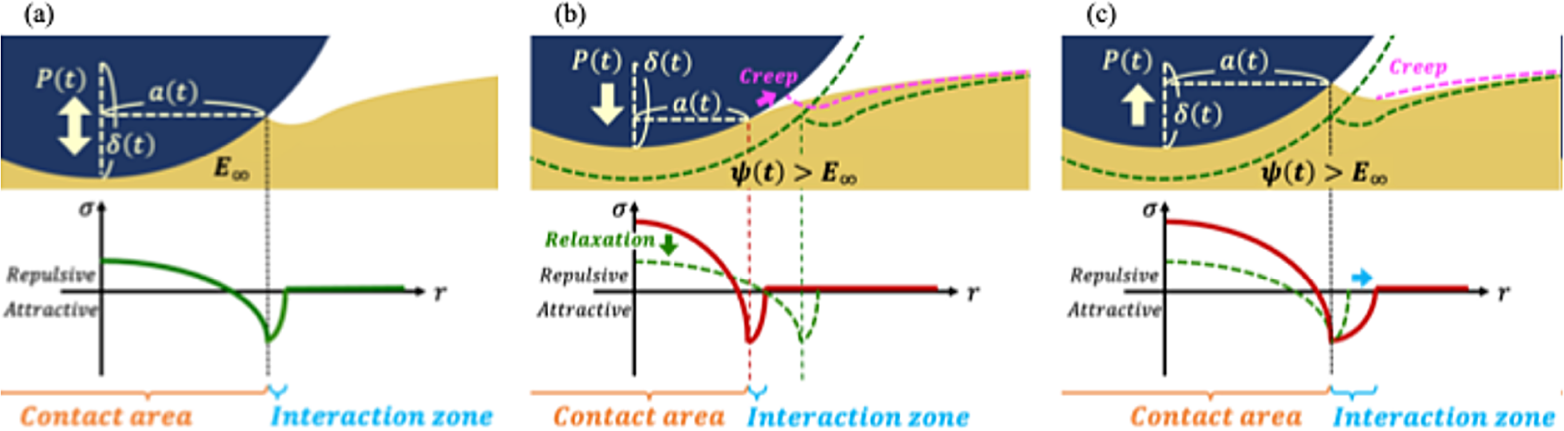
Schematic diagrams of contact situations. (a) shows the loading and unloading situation under a fully relaxed state. (b) and (c) respectively show the loading and unloading situations where relaxation is in progress.

As can be seen in Figure 3a, for the 10 nm/s curve, the contact radius estimated from the Barthel model agreed well with those from the JKR model (and thus from the MD model, since there was little difference between the JKR and the MD models in this case, as mentioned in Supporting Information). Since this force curve was measured at a slow enough rate to be handled by the elastic model in a fully relaxed state, it is reasonable that almost the same results can be obtained when analyzed with the Barthel model. A schematic diagram is shown in Figure 4a. The contact situation proceeds in a fully relaxed state, so it is the relaxed modulus *E*_∞_ that dominates. Note that although the interaction zone is depicted large in the figure, it is actually very small (which is why the contact radius matches in the Barthel and JKR models). Additionally, a minor difference was found at point A, where the Barthel model estimates a smaller contact radius. Even with a sufficiently slow ramp rate, there should be somewhat instantaneous tip movement at the jump-in, so it is not surprising that the contact radius there is smaller than expected from the elastic model. In fact, the Barthel model may be more accurate in estimating the contact, given that it can represent such instantaneous behavior at the jump-in point.

In Figure 3b, for the 9770 nm/s curve, the contact radius estimated from the Barthel model differed significantly from those from the JKR model. First, the contact radius for the loading was estimated to be about 10 nm smaller than in the JKR model. This behavior seems reasonable, as the PDMS relaxation upon contact should be insufficient as the ramp rate increases. A schematic diagram is shown in Figure 4b. Under fast ramp rates, the PDMS at the moment of contact is in the process of relaxing, so the relaxation function takes a value larger than *E*_∞_. Of course, the PDMS relaxes from there, but at each moment of contact, there is a component that has not reached fully relaxed state, so the contact radius should be smaller than in the JKR model, in line with the penetration being smaller. Indeed, as shown in Figure 3c, the penetration at 9770 nm/s is overall smaller than at 10 nm/s. Second, just after the beginning of the unloading phase, a region where the contact radius did not change much was identified, which was not seen in the JKR analysis. This region is referred to as the “stick region” in Barthel’s articles.^36,37^ It can be inferred from Figure 3d that this sticking behavior has a rate dependency, since the “stick region” becomes more pronounced as the rate increases. This means that the “stick region” is due to viscoelasticity, so it is not surprising that it does not appear in the JKR analysis. The contact radius in unloading changed more slowly than in the JKR model, not only in the “stick region” but also in subsequent regions. As a result, the contact radius estimated at point B is about 10 nm smaller than the value estimated from the JKR model, but at point C, it is about 10 nm larger than the JKR model.

**Figure 5:**
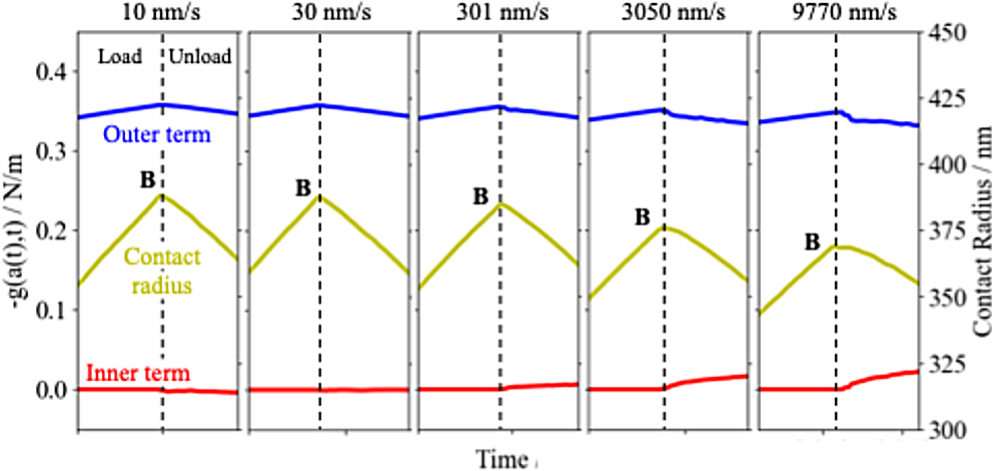
Components of *g*(*a*(*t*),*t*) in the vicinity of the “stick region” for various ramp rates. The red and blue lines represent components originating from the contact area and the interaction zone, respectively. The yellow lines represent the contact radius.

To discuss such behaviors in more detail, the components of *g*(*a*(*t*),*t*) around the “stick region” were investigated. As mentioned earlier, in eq 22, *g*(*a*(*t*),*t*) can be decomposed into the first term derived from the adhesive stress inside the interaction zone (i.e., outer term) and the second term derived from the stress inside the contact area (i.e., inner term). Based on this, Figure 5 shows the *g*(*a*(*t*),*t*) in the vicinity of the “stick region”, divided into the outer term (blue line) and the inner term (red line), with the contact radius (yellow line), for various ramp rates. In the case of loading, the inner term is zero since *t* = *t*_–_ in eq 22, thus *g*(*a*(*t*),*t*) is always dominated by the outer term, that is, the interaction zone. In the case of the unloading, on the other hand, the inner term remains zero at small rate, while at large rates the inner term also affects *g*(*a*(*t*),*t*) to some extent. The inner term represents the stress relaxation that has taken place from the moment *a*(*t*) enters inside the contact area to the moment it moves out again. The fact that the inner term is zero at sufficiently small rate implies that the stress relaxation has already completed by the moment the point *a*(*t*) enters the inside of the contact area, which corresponds to Figure 4a. By contrast, a nonzero value of the inner term at large rates implies that the stress relaxation is insufficient by the moment the point *a*(*t*) enters inside the contact area, and that the stress relaxation during the contact is not negligible, as illustrated in Figure 4b,c. It can therefore be suggested that the tendency of the contact radius during loading to be smaller at higher rates, as shown in Figure 3d, results from the fact that the inside of the contact area is in the process of relaxation. Moreover, Barthel et al. pointed out that the “stick region” also originates from the stress relaxation inside the contact area during contact.^36^ If the unloading process is started in an incomplete state of relaxation as in Figure 4c, part of the applied tensile stress is canceled out by the stress relaxation still ongoing inside the contact area, so that the contact radius is less likely to change in response to the applied tensile stress, i.e., the sticking behavior occurs. In the present analysis, as shown in Figures 3d and 5, the stick region is indeed more pronounced as the contact radius during loading becomes smaller and the inner term becomes larger at higher rates.

The discussion so far suggests that the viscoelastic behavior inside the contact area does contribute to the rate dependence of the contact state. Consequently, it is not appropriate to assume that the interior of the contact area is in a completely relaxed state, as in the “quasi-viscoelastic” models, at least in systems like the present one where the loading process affects the unloading process. Meanwhile, it has been pointed out that the interaction zone also contributes to the rate dependency in many papers. Therefore, the rate dependence of the interaction zone is examined in the following section.

### The Interaction Zone and the Apparent Work of Adhesion

**Figure 6:**
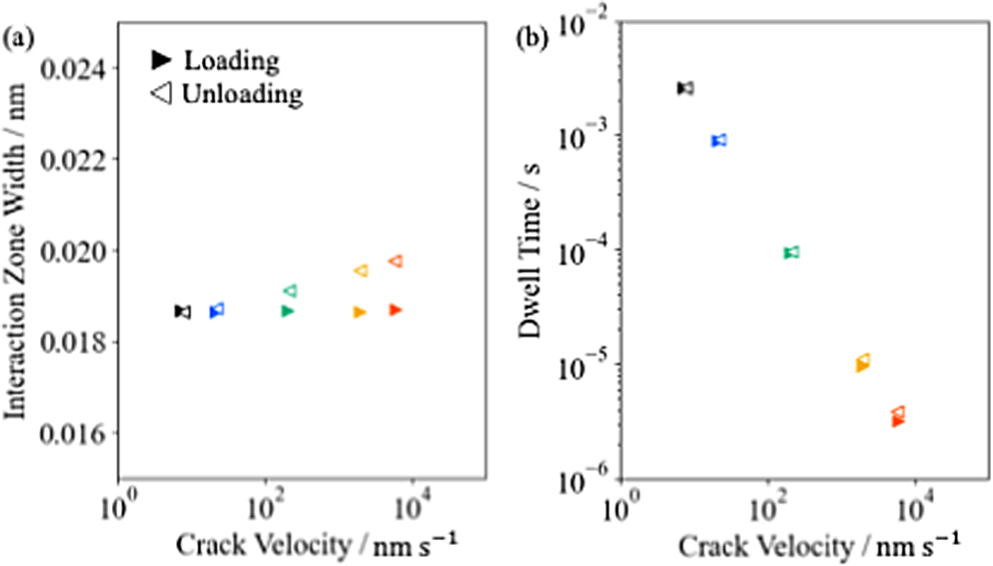
Crack velocity dependence of (a) the interaction zone width and (b) the dwell time.

The interaction zone is described by its width ε(*t*) and the dwell time *t*_r_(*t*), as mentioned earlier. Figure 6a,b show these values against the moving velocity of the contact line (|d*a*(*t*)/d*t*|, referred to as the crack velocity), respectively. Note that these values can be obtained at each time of each force curve, so their average values are plotted. The interaction zone width was almost constant regardless of the crack velocity during loading, whereas it tended to become slightly wider with increasing the crack velocity during unloading. In response to this, the dwell time was slightly smaller for loading at higher crack velocities.

The rate dependence of the interaction zone width has an inseparable relationship with the crack shape in the vicinity of the contact line.^24−27^ The mechanical properties near the contact line and the creep deformation that occurs there determine this relationship. In the case of loading, the creep deformation near the contact line directly infulences the crack shape (Figure 4b). Therefore, it is both the mechanical properties and the creep behavior near the contact line that contribute to the interaction zone. In the case of unloading, the creep deformation near the contact line is also present but does not affect the interaction zone, as the creeping region almost immediately goes outside the interaction zone (Figure 4c). Therefore, it is only the mechanical properties near the contact line that define the interaction zone during unloading. This difference is considered to be reflected in the difference between loading and unloading in Figure 6a.

The apparent work of adhesion can also be discussed from this point on. It has been shown that the apparent work of adhesion based on the Barthel model can be expressed as follows.^38^ For loading, the energy required to increase the contact radius is apparently reduced because the creep deformation that occurs in the vicinity of the contact line has the effect of increasing the contact area (Figure 4b). By incorporating such creep deformation effects, the following equation can be obtained.
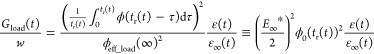
25*G*_load_(*t*) is the apparent work of adhesion for loading at *t*, ε_∞_(*t*) is the interaction zone width under fully relaxed modulus, and ϕ_0_(*t*_r_(*t*)) is a moment of the creep compliance that links the apparent work of adhesion to the creep deformation in the vicinity of the contact line. Given that the interaction zone was almost unchanged during loading in Figure 6a, it can be said that the creep deformation component ϕ_0_(*t*_r_(*t*)) is the main cause of the change in the apparent work of adhesion in the present system. For the unloading, creep deformation has no effect on the reduction of the contact radius as described above. What does affect is the interaction zone dominated by the mechanical properties near the contact line. If the deformation near the contact line is more instantaneous and the relaxation there is insufficient (i.e., behaves stiffly), the contact state can be said to shift in the DMT direction in the JKR–DMT transition. Thus, it widens the interaction zone (Figures 4c and 6a) and increases the apparent work of adhesion. That is
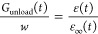
26

**Figure 7:**
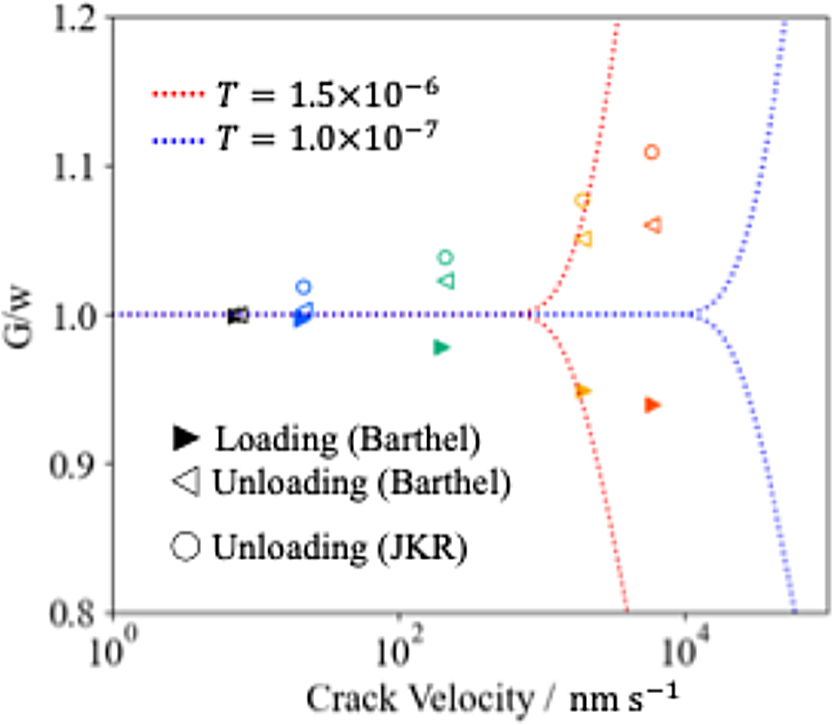
Crack velocity dependence of the apparent work of adhesion estimated from the Barthel (triangles) and JKR models (circles). The dashed lines represent the Greenwood approximation.

The apparent work of adhesion was calculated based on eqs 25 and 26, and plotted against the crack velocity as shown in Figure 7. An increase in the apparent work of adhesion at unloading and a decrease at loading with increasing the crack velocity can be observed. Therefore, the apparent work of adhesion also has an effect on the rate dependence of the contact state in the present force curves, together with the viscoelastic behavior inside the contact area discussed earlier. Note that, the crack velocity dependence of the work of adhesion obtained from the JKR model for unloading was estimated to be about 5% larger than that of the Barthel model. This means that even if the apparent work of adhesion is obtained from the unloading curve which appears to be able to be fitted with the JKR model successfully, it may deviate from the accurate apparent work of adhesion.

Greenwood has numerically calculated the rate dependence of the apparent work of adhesion for both loading and unloading, and proposed approximate equations.^25,26^ In these equations, the apparent work of adhesion under the SLS model with a relaxation time *T* is represented via the interaction zone width. Based on this, the rate dependence of the apparent work of adhesion obtained by Greenwood’s method with *T* = 1.5 × 10^–6^ s and *T* = 1.0 × 10^–7^ s was calculated and displayed in Figure 7 (red and blue dashed lines). The slope of the crack velocity dependence obtained from the Greenwood model was steeper than the present Barthel analysis results. When *T* is changed, the graph shifted along the velocity axis, but the slope did not change and therefor did not agree with the results of the Barthel analysis. The slope in the Greenwood model is dominated by the SLS model used, which means that the same slope will be obtained under the same SLS model. The fact that the rate dependence of the apparent work of adhesion obtained from the Barthel analysis is gentler than that from the Greenwood model may indicate the limitations of expressing the rate dependence of contact based on the SLS model. As mentioned in Supporting Information, the Barthel analysis in this study is implemented by representing viscoelasticity using the SLS model, with its relaxation time is optimized for each force curve. In this context, introducing viscoelastic models that are more representative of real elastomer behavior, such as the Prony approximation or fractional viscoelastic models,^55^ into the Barthel model could be of interest for future studies.

It is also suggested in Figure 7 that the force curve measurements carried out in this study were in the very low velocity range. Greenwood noted that the above approximate equations tend to overestimate the slope of the velocity dependence in the very low velocity range. It has been pointed out that the Barthel model can better represent the velocity dependence in such a very low velocity range.^38^ Therefore, this perspective could also be the reason for the deviation from the Greenwood model and the Barthel analysis results. Analysis of force curves acquired at higher ramp rate may provide a better understanding of this aspect.

## Conclusion

The force curves of the elastomers measured by AFM were analyzed using the Barthel model and compared with the results from the elastic JKR and MD models. The PDMS force curves showed elastic behavior at very low ramp rates, but as the ramp rate increased, viscoelasticity became non-negligible, and analysis with the elastic contact model was no longer appropriate, necessitating analysis with the Barthel model, which takes viscoelasticity into account. Even in the analysis of elastic force curves, the Barthel analysis may be more accurate regarding the jump-in point, which actually behaves instantaneously.

The analysis via *g*(*a*(*t*),*t*) suggested that viscoelastic behavior, both inside the contact area and the interaction zone, influences the contact state at higher ramp rates. In conventional studies dealing with viscoelastic contact, it is often assumed that the interior of the contact area is in a perfectly relaxed state, but the present analysis shows that this assumption is not always true. The viscoelastic behavior inside the contact area affected the reduction of the contact radius during loading and the formation of a “stick region” during unloading, while the viscoelastic behavior inside the interaction zone affected the apparent work of adhesion during loading and unloading. It can now be said that the “stick region” and the apparent work of adhesion in actual force curves can be quantitatively analyzed using the Barthel analysis.

In implementing the Barthel model, the viscoelasticity was represented by the SLS model based on a single relaxation time. However, as the actual elastomer behavior is explained by multiple relaxation times, there may be limitations in representing contact based on the SLS model. Indeed, while the contact state of the PDMS was successfully analyzed in this study, the contact state of the SBR was more complex and has not been analyzed yet. Therefore, it is a subject for future work to analyze complex viscoelastic contacts by introducing more realistic viscoelastic models that are even closer to practical elastomers or by considering microscopic roughness. Beyond these topics, potential applications include the analysis of the mechanical properties of polymer blends and nanocomposites. To achieve this, further improvements in lateral resolution using smaller AFM tips will also be an important focus for future research.

## Supporting Information Available

The details of the analytical approaches using the elastic contact models (the JKR and the MD models) and the viscoelastic contact model (the Barthel model) are described (PDF)
