# Supplementary material for: Atomic Force Microscopy Analysis of Velocity Dependent Adhesive Viscoelastic Contact
Source: Langmuir. 2024 Nov 8;40(46):24565–75. doi: 10.1021/acs.langmuir.4c03370 (PMC11580373; doi:10.1021/acs.langmuir.4c03370)
Supplement: Supplementary file 1 — la4c03370_si_001.pdf [file la4c03370_si_001.pdf]

Atomic Force Microscopy Analysis of Velocity Dependent Adhesive Viscoelastic Contact

Nobuhito Onozuka[1] and Ken Nakajima[1,*]

[1]School of Materials and Chemical Technology, Tokyo Institute of Technology, 2-12-1, O-Okayama, Meguro-ku, Tokyo, 152-8552, JAPAN

SUPPORTING INFORMATION

1. Analytical Approaches

The Elastic Contact Model

If the force curve can be regarded as an elastic contact, the analysis can be performed with an elastic contact model. Since it has been found that the DMT model tends to have a larger error due to ignoring deformation caused by adhesion, especially in soft materials[1], the JKR model or intermediate models in proximity to JKR limit may be preferred. In this paper, force curve analyses with the JKR and MD models were performed. In these theories, it is Young's modulus E and the work of adhesion w that determine the contact situation. Being an elastic contact, i.e., the contact state is in equilibrium, it is assumed that E and w take constant values throughout the entire contact. Taking into account the difficulty in determining the zero-point of δ, estimation of the contact radius a was conducted through an optimization method. The optimization calculations were performed using the \`minimize\` function of the \`optimize\` module provided in the Python Scipy package.

For analysis using the JKR model, the following procedure was used (Figure S1(a)).

① First, the unloading curve was analyzed. Since the unloading curve usually has more data points than the loading curve, and many existing examples of force curve analysis use the unloading curve, it is fair to start with an analysis of the unloading curve. Given E and w_{unload} as fitting parameters, P and δ were calculated using Equations (7) and (8) with various a. Then, the parameters were optimized to minimize the error between this theoretical P and δ and the experimental P and δ for the unloading curve. At this point, all experimental δ for unloading were offset so that at a specific point (here, the jump-out point C), its penetration δ_C matches between calculated and theoretical values. Offsetting δ in this manner means considering that all points of the unloading curve can be represented by the JKR model. The E optimized from the unloading curve was assumed to dominate the entire contact. All a for unloading curve were

then calculated from Equation (7) using the optimized E and w_{unload}.

② Second, the loading curve was analyzed using the optimized E. Given w_{load} as a fitting parameter, the optimization was performed similarly to the unloading curve. This time, all experimental δ for loading were offset so that at the maximum load point B, its contact radius a_B (which can be obtained using Equation (7) with w_{unload} and w_{load} for unloading and loading, respectively) matches between loading and unloading. This is because the contact radius should be continuously connected at point B, where the transition between the loading phase and unloading phase occurs. All a for loading curve were calculated from Equation (7) using the optimized E and w_{load}.

Note that δ offset and w are obtained separately for the loading and unloading curves.

For analysis using the MD model, the procedure proceeded in the same manner as for the JKR analysis (Figure S1(b)).

① First, the unloading curve was analyzed. Given λ_{unload} and w_{unload} as fitting parameters, P and δ were calculated using Equations (10) to (13) with various m. Then, those parameters were optimized to minimize the error between the theoretical P and δ and the experimental P and δ for the unloading curve, with the experimental δ being offset accordingly using point C. From λ_{unload} and w_{unload} optimized for the unloading curve, Young's modulus E can be obtained from Equation (10). As in the JKR analysis, this E obtained from the unloading curve was assumed to dominate the entire contact. All a for unloading curve were then calculated from Equation (13) using the optimized λ_{unload} and w_{unload}.

② Second, the loading curve was analyzed using the optimized E. Given λ_{load} as a fitting parameter, the optimization was performed in the same manner as described above. All experimental δ for loading were offset so that the contact radius is continuously connected across point B. From λ_{load} optimized for the loading curve, w_{load} can be obtained from Equation (10). All a for the loading curve were calculated from Equation (13) using the optimized λ_{load} and w_{load}.

In the MD model, in addition to the δ offset and w, m, which defines the interaction zone, is obtained separately for the loading and unloading curves. Again, if the two curves completely overlap, then all values should be the same for the loading and unloading curves.

Although the above procedures are slightly cumbersome, they are effective for comparing the elastic contact models with the viscoelastic Barthel model later. This procedure can treat the work of adhesion for loading and unloading independently, as seen in viscoelastic contacts, while keeping Young's modulus constant. At any rate, δ offset and w will result in same values for the loading

and unloading curves if the two curves completely overlap, i.e., in the case of elastic contact.

Conventionally, when applying the JKR model to force curve analysis, algebraic approaches[1,2,3] or curve fitting and parametric fitting[4,5] utilizing characteristic points on the force curve (e.g. the jump-out point C) were used. The present procedure for the JKR analysis is also a kind of parametric fitting, but it is unique in that E and w are obtained for loading and unloading respectively, taking into account the arbitrariness of the zero-point of the penetration and the continuity of the contact radius. It is also an effective method in that it can be applied in the same way to the MD models, which are conventionally difficult to handle. Furthermore, the deviation of the force curve from elastic contact theories can be directly discussed as a fitting error, which makes these scalable methods.

<u>The Viscoelastic Contact Model</u>

In describing the Barthel model, the viscoelasticity of the sample must be represented by a viscoelastic model. Here, a simple standard linear solid (SLS) model was employed, and the relaxation function and the creep compliance function were expressed respectively as follows:

$$\psi(t) = \left(\frac{1}{k} + \left(1 - \frac{1}{k}\right) e^{-\frac{kt}{T}}\right) \frac{{E_0}^*}{2}, \tag{S1}$$

$$\phi(t) = \left(1 - \left(1 - \frac{1}{k}\right) e^{-\frac{t}{T}}\right) \frac{2}{{E_\infty}^*}. \tag{S2}$$

$k \equiv {E_0}^*/{E_\infty}^*$, where E_0 is the instantaneous modulus and E_∞ is the relaxed modulus. T is the relaxation time that governs the time scale of the relaxation phenomena. E_∞ can be estimated in the analysis as discussed below, but E_0 and T cannot. Therefore, in the analysis, E_0 was given a fixed value, and T was treated as a fitting parameter.

Force curve analysis using the Barthel model was performed using the following procedure (Figure S1(c)). The integral calculations were performed with the `integrate` module provided in Python SciPy package.

① Using a force curve that can be regarded as an elastic contact, w and E_∞ were calculated based on the JKR analysis as described in the previous section. Since this force curve was measured at such a low speed that it can be considered elastic, the obtained modulus can be considered as the relaxed modulus. As will be discussed later, the samples used in this study were estimated to have sufficiently small interaction zones. Therefore, w and E_∞ obtained by the JKR fitting are reasonable.

② Given the contact radius a_A at the jump-in point (point A) as a fitting parameter, δ_A and E_A

were calculated from Equations (7) and (8) using w and P_A. E_A can be considered as the modulus governing the contact at the jump-in point. This process is intended to incorporate the relaxation state at the jump-in point, assuming that the contact state at there can be expressed by the JKR model. In this way, depending on the size of a_A given (and δ_A calculated), the relaxation condition at point A (which is reflected in E_A) can be captured into the force curve analysis. All experimental δ (for loading and unloading) were then offset so that at the jump-in point, its penetration δ_A matches between calculated and experimental values.

③ All $a(t)$ were calculated considering the following steps.

A) Suppose the contact radius is known at times up to t_{n-1}, i.e., (t_A, P_A, δ_A, a_A), (t_1, P_1, δ_1, a_1), (t_2, P_2, δ_2, a_2),…, ($t_{n-1}, P_{n-1}, \delta_{n-1}, a_{n-1}$) are all known. Here, the time at point A is taken as the starting point of the contact, i.e., $t_A = 0$.

B) The a_n at time t_n can be obtained by optimizing in a way that minimizes the difference between the experimental value of P_n and the calculated value of P_n from Equation (19). As noted above, E_0 was given a fixed value and T was treated as a fitting parameter.

The above steps can be applied inductively to all points in turn if a_A and δ_A are known. Therefore, the process up to this point allows the calculation of all $a(t)$ at each point of the force curve. Note, however, that the $a(t)$ calculated here considers only Equation (19) and does not consider the self-consistency equations.

④ Using the self-consistency equations, all $\epsilon(t)$ and $t_r(t)$ can be obtained. Note that since $a(t)$ has already been determined above, the crack velocity $da(t)/dt$ can be calculated at each time. Then Equation (20) (or (21)) and $t_r(t) = \epsilon(t)/|da(t)/dt|$ can be solved as a simultaneous equation for $\epsilon(t)$ and $t_r(t)$.

⑤ $a(t)$, $\epsilon(t)$, and $t_r(t)$ calculated so far must concurrently satisfy the coupling equations. To check this, the auxiliary function $g(a,t)$ was calculated from Equations (22) and (23), and then it was verified whether they were consistent. The fitting parameters, a_A and T, were optimized until the two $g(a(t),t)$ became consistent.

At first glance, this fitting method may appear to be incomplete, using only a small part of the force curve. However, the advantage of the Barthel's equation, such as Equation (19), is that when calculating $a(t)$ at a certain point, it incorporates the history up to that point, meaning all the points on the force curve leading up to that point are considered. Furthermore, the auxiliary function $g(a,t)$ was calculated from Equations (22) and (23), and the fitting parameters were optimized so that the difference between these $g(a,t)$ values is minimized across the entire force curve. Therefore, it does not mean that only a part of the force cure is being used.

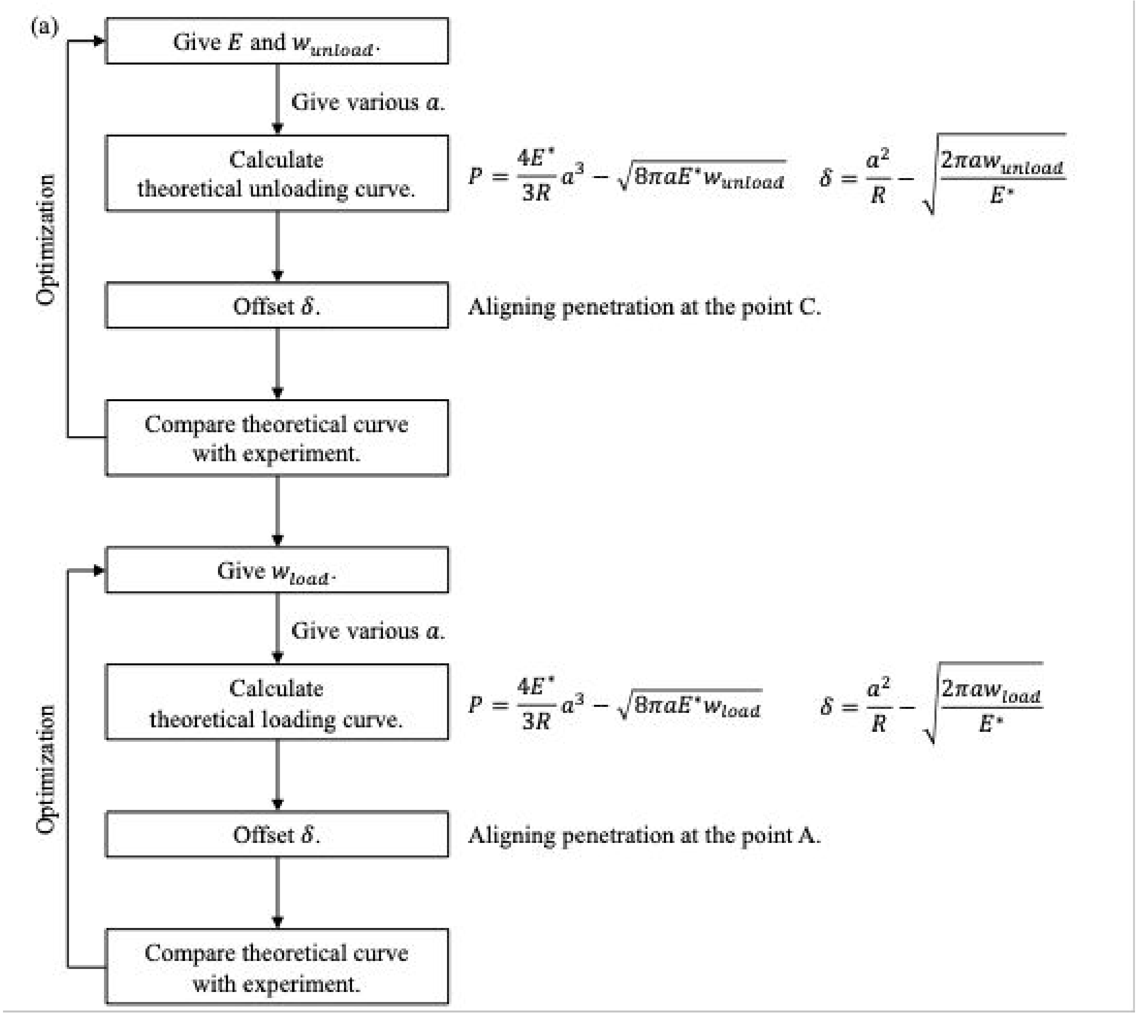
(a)
Give E and w_{unload}.
Give various a.
Calculate theoretical unloading curve.
$P = \frac{4E^*}{3R}a^3 - \sqrt{8\pi a E^* w_{unload}}$
$\delta = \frac{a^2}{R} - \sqrt{\frac{2\pi a w_{unload}}{E^*}}$
Offset δ.
Aligning penetration at the point C.
Compare theoretical curve with experiment.
Optimization
Give w_{load}.
Give various a.
Calculate theoretical loading curve.
$P = \frac{4E^*}{3R}a^3 - \sqrt{8\pi a E^* w_{load}}$
$\delta = \frac{a^2}{R} - \sqrt{\frac{2\pi a w_{load}}{E^*}}$
Offset δ.
Aligning penetration at the point A.
Compare theoretical curve with experiment.
Optimization

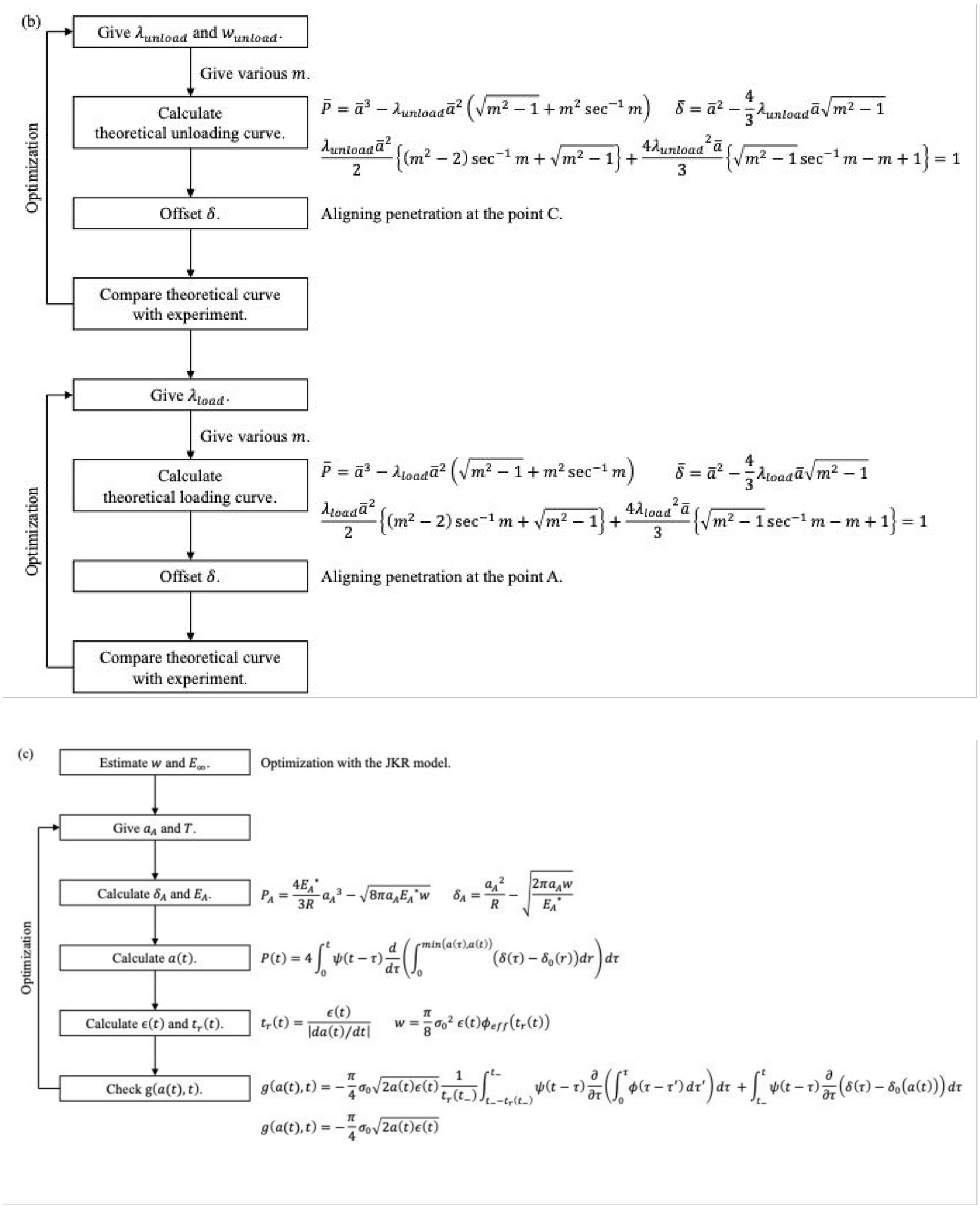

Figure S1 Analysis procedures with (a) the JKR model, (b) the MD model, and (c) the Barthel model.

2. Force curve analysis with the elastic JKR and MD models

The force curve of the PDMS at a sufficiently small ramp rate can be regarded as elastic because of the overlap of the loading and unloading curves. So, first of all, such an elastic curve at 10 nm/s

was analyzed using the JKR and MD models. Figure S2(a) shows the time variation of the contact radius obtained from the JKR and MD models, and Table S1 shows the physical values estimated from them. In terms of comparing the JKR and the MD models, the physical values estimated from those two models in Table S1 were almost identical, and the estimated contact radius in Figure S2(a) entirely overlaps. The work of adhesion w obtained from the loading and unloading curves was almost same in both models, as expected for the elastic contact. Since the parameter λ for the MD model was about 117, implying that the contact was quite JKR-like, it seems reasonable that there was almost no difference between the two models. The interaction zone size ϵ was estimated to be about 0.01 nm throughout the contact, which was quite small compared to the contact radius. Figure S3(a) shows the experimental force curves during contact reflecting δ offset for the loading and unloading, with the theoretical curves obtained from the JKR model. Same figure for the MD model was omitted as no differences were found with the JKR model. The experimental curves and the theoretical curves were in good agreement for the loading and unloading, confirming that the analysis was carried out correctly. Note that the penetration at point B continuously connected between the loading and unloading (Figure S3(a) inset).

Next, although somewhat unreasonable, the force curve at a higher ramp rate (9770 nm/s), where its viscoelasticity was not negligible, was analyzed using the JKR and MD models. The results are shown in Figure S2(b), Figure S3(b), and Table S1. Again, there was little difference between the results from the JKR model and the MD model. In Figure S3(b), while the unloading curve agreed well with the experimental curve and the theoretical curve, the loading curve showed a large mismatch between the two. The present analysis assumes that the same Young's modulus (i.e., the slope of the force curve) dominates in both the loading and unloading phases (and this assumption seems at least reasonable for the bulk properties). However, when the ramp rate is large, the loading and unloading curves do not overlap, i.e., the slopes are different, so it becomes unfeasible to analyze the loading curve with the slope of the unloading curve. Due to this, in minimizing the error between the theoretical curve and the experimental curve during loading, the penetration at point B became discontinuous with the unloading curve (Figure S3(b) inset). Therefore, though the time variation of the contact radius in Figure S2(b) appears to be satisfactory, the validity of this result is questionable. As for Table S1, the results of the loading analysis are unreliable, while E and w_{unload} from the well-executed unloading analysis may be reasonable.

As described above, treating viscoelastic force curves with elastic contact theories faces limitations, especially when discussing the contact radius. Some papers discuss the contact radius of the unloading phase with elastic models after careful preparation of an experimental system in which the start of

unloading (i.e. point B) can be regarded as a fully relaxed state[6-9]. However, it is not guaranteed that point B is in a fully relaxed state in AFM force curve measurements. In addition, the loading and unloading curves cannot be treated in a unified manner under experimental systems specializing in unloading analysis. Therefore, to correctly discuss the contact radius in both loading and unloading of viscoelastic force curves, an analysis based on the viscoelastic contact model is required.

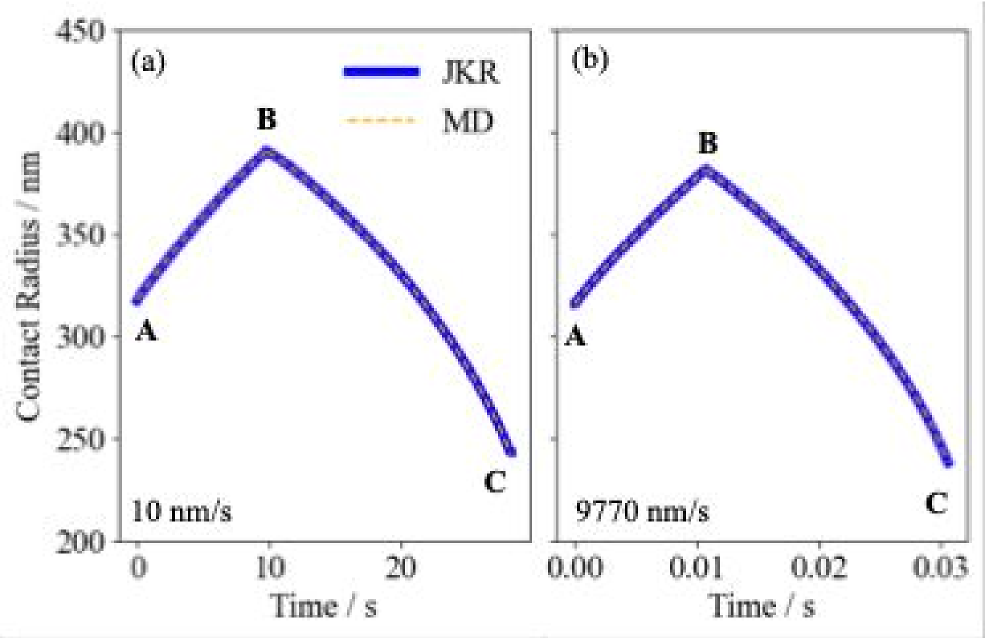

Figure S2 The time variation of the contact radius for the ramp rate of (a) 10 nm/s and (b) 9770 nm/s. Thick solid blue lines and thin dashed orange lines represent the contact radius from the JKR and MD models, respectively.

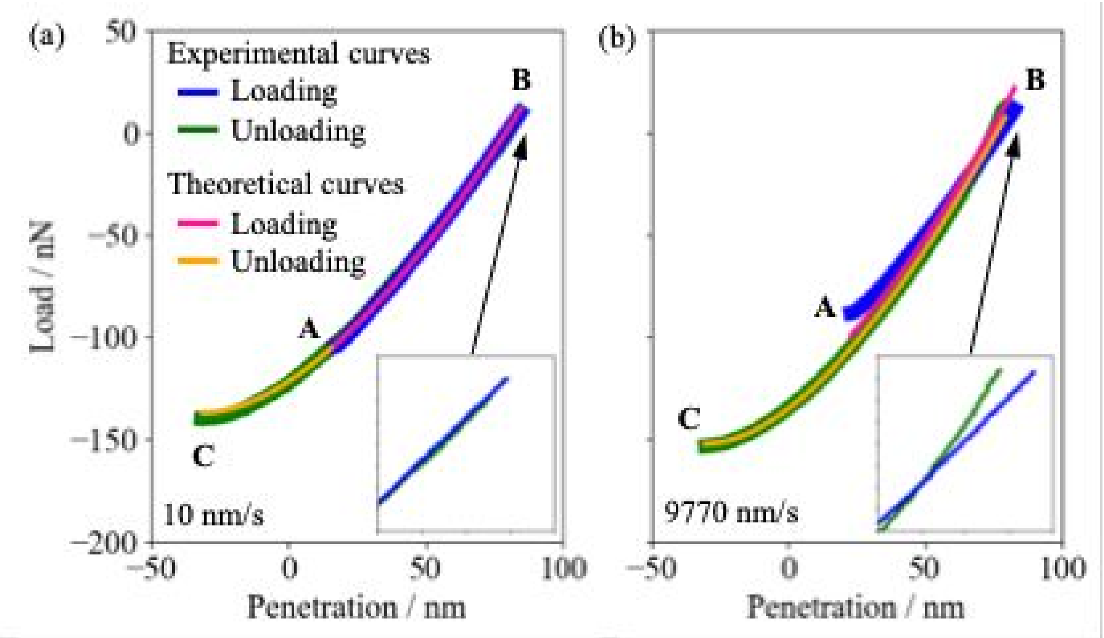

Figure S3 Comparison between experimental curves (blue lines for loading and green lines for unloading) and theoretical curves (red lines for loading and orange lines for unloading). (a) shows curves for 10 nm/s, while (b) shows curves for 9770 nm/s. Insets provide an enlarged view around point B.

Table S1 Physical values estimated in force curve analysis using the JKR and MD models.

	Elastic curve (10 nm/s)				Viscoelastic curve (9770 nm/s)			
	JKR		MD		JKR		MD	
	Loading	Unloading	Loading	Unloading	Loading	Unloading	Loading	Unloading
E / MPa	3.38		3.36		3.96		3.97	
w / Nm^{-1}	0.0467	0.0466	0.0465	0.0465	0.0493	0.0517	0.0494	0.0518
λ	-	-	117.7	117.7	-	-	109.5	113.1

3. Force curve analysis with the viscoelastic Barthel model

Following the discussion so far, analyses based on the Barthel model were conducted using the method shown above. E_∞ and w were determined to be 3.38 MPa and 0.0466 N/m, respectively, based on the JKR analysis of the force curve of 10 nm/s in Table S1. E_0 was assumed to be 1 GPa, a common value for the instantaneous modulus of elastomers. In the optimization, optimal T and a_A were grid-searched in the range of 1.0×10^{-8} to 5.0×10^{-6} s and 175 to 350 nm, respectively, such that the auxiliary function $g(a(t),t)$ obtained from Equations (22) and (23) was consistent

throughout the entire time period of the contact. Recall that Equations (22) and (23) both correspond to the normal surface stress acting on the surface, but Equation (22) is an expression as a superposition of the history of stress acting on the location of interest, while Equation (23) is an expression of the stress currently acting on that location based on the double-Hertz model. Therefore, under optimal T and a_A, $g(a(t),t)$ obtained from Equations (22) and (23) should be equal. As an example, consider the analysis of the force curve at 301 nm/s. Under the optimal T and a_A, $g(a(t),t)$ obtained from Equation (22) (thick solid blue line) and that obtained from Equation (23) (thin dashed orange line) were consistent over the entire time period, as shown in Figure S4(a). Note that, although $g(a(t),t)$ is defined as a transform of negative adhesive stress, its sign is reversed in this figure for clarity. To achieve such an optimal result, the difference between two $g(a(t),t)$ curves (Δg) was examined and mapped for various T and a_A as shown in Figure S4(b). The point where the minimum Δg was obtained in this heat map (X sign) was considered to be the optimum values.

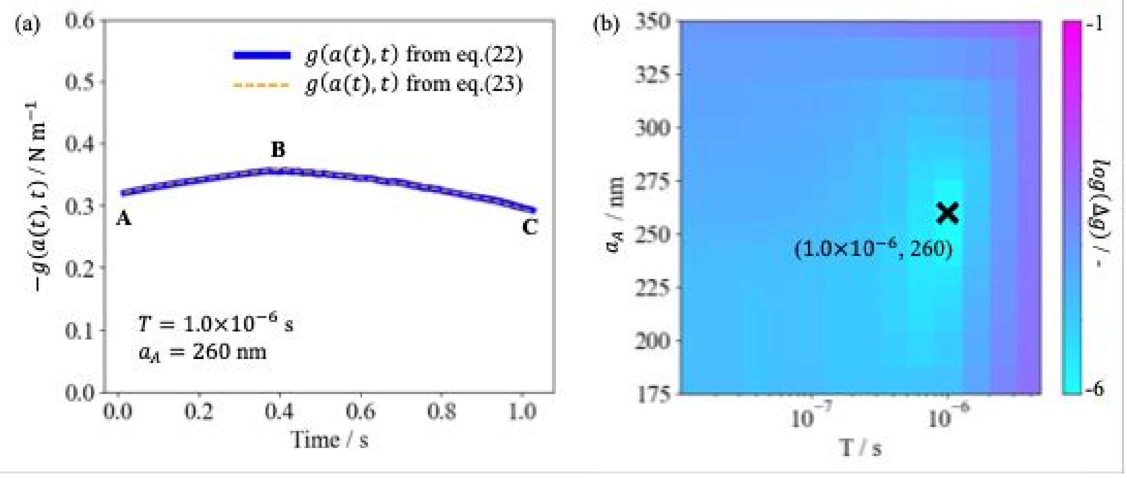

Figure S4 The Barthel analysis of the force curve at 301 nm/s. (a) A comparison of two $g(a(t),t)$ from Equations (22) and (23) at optimal T and a_A. (b) Δg mapped for different T and a_A.

The optimized fitting parameters are shown in Table S2. As the ramp rate increased, the optimal value of a_A became steadily smaller. a_A was introduced to capture the relaxation state at the jump-in point A, which was reflected in E_A. Thus, this result implies that a_A gets smaller and E_A gets larger as the jump-in behavior becomes more instantaneous under the large ramp rates. This is consistent with the rate dependency in Figure 2(a), where the tip was less likely to be pulled in at point A at higher rates. T was estimated to be of the very small order of 10^{-6} to 10^{-7} s. Since T obtained here is a time constant that approximates the actual PDMS relaxation behavior in a simple SLS model with a single relaxation time, its detailed physical meaning is not entirely clear. Yet, it can be assumed to have some relevance to the actual relaxation behavior of the PDMS. Given the small

tanδ of PDMS, it seems reasonable that T is very small and that the relaxation completes almost instantaneously. It is of interest that T tended to decrease as the ramp rate increased. As T is an intrinsic property of the PDMS in the SLS model, it should inherently be estimated at a constant value. The fact that T was not constant in the present analysis may indicate that the relaxation process contributing to the contact phenomena may vary according to the time scale of the measurement, as the actual elastomer relaxation is expressed by multiple relaxation phenomena. This perspective needs better understanding in the future, for example by considering the Barthel analysis using more realistic viscoelastic models such as the Prony approximation or fractional viscoelastic models[10]. Another aspect that may need to be investigated is the possibility that the measured force curves may reflect not only the physical properties of the PDMS, but also other factors such as the stiffness of the AFM probe and its responsiveness.

Table S2 Optimized fitting parameters for the Barthel analysis.

Ramp rates / nm s^{-1}	10	30	301	3050	9770
T / s	1.5×10^{-6}	1.5×10^{-6}	1.0×10^{-6}	2.5×10^{-7}	1.0×10^{-7}
a_A / nm	305	290	260	255	250
E_A / MPa	3.58	4.98	6.27	7.16	7.87

References

(1) Fujinami, S.; Ueda, E.; Nakajima, K.; Nishi, T. Analytical methods to derive the elastic modulus of soft and adhesive materials from atomic force microscopy force measurement. *J. Polym. Sci., Part B: Polym. Phys.*, **2019**, 57 (18), 1279-1286.

(2) Sun, Y.; Akhremitchv, B.; Walker, G.C. Using the adhesive interaction between atomic force microscopy tips and polymer surfaces to measure the elastic modulus of compliant samples. *Langmuir*, **2004**, 20 (14), 5837-5845.

(3) Grunlan, J.C.; Xia, X.; Rowenhorst, D.; Gerberich, G.G. Preparation and evaluation of tungsten tips relative to diamond for nanoindentation of soft materials. *Rev. Sci. Instrum.*, **2001**, 72 (6), 2804-2810.

(4) Dokukin, M.E.; Sokolov, I. Quantitative mapping of the elastic modulus of soft materials with HarmoniX and PeakForce QNM AFM modes. *Langmuir*, **2012**, 28 (46), 16060-16071.

(5) Ebenstein, D.M.; Wahl, K.J. A comparison of JKR-based methods to analyze quasi-sitatic and

dynamic indentation force curves. *J. Colloid Interface Sci.*, **2006**, 298 (2), 652-662.
(6) Violano, G.; Chateauminois, A.; Afferrante, L. A JKR-like solution for viscoelastic adhesive contacts. *Front. Mech. Eng.*, **2021**, 13 (7), 2021.
(7) Violano, G.; Afferrante, L. Adhesion of compliant spheres: an experimental investigation. *Proc. Struct. Integr.*, **2019**, 24, 251-258.
(8) Das, D.; Chasiotis, I. Rate dependent adhesion of nanoscale polymer contacts. *J. Mech. Phys. Solids*, **2021**, 156, 104597.
(9) Ciavarella, M.; Joe, J.; Papangelo, A.; Barber, J.R. The role of adhesion in contact mechanics. *J. R. Soc. Interface*, **2019**, 16 (151), 20180738.
(10) Bonfanti, A.; Kaplan, J.L.; Charras, G.; Kalba, A. Fractional viscoelastic models for power-law materials. *Soft Matter*, **2020**, 16, 6002-6020.
